# Frictional Losses of Polyamides Mounted on Tensioning Guides in Contact with Chains

**DOI:** 10.3390/ma15041345

**Published:** 2022-02-11

**Authors:** Radu Gabriel Velicu, Mihai Tiberiu Lates, Cornel Catalin Gavrila

**Affiliations:** Faculty of Product Design and Environment, Transilvania University of Brasov, 500036 Brașov, Romania; latesmt@unitbv.ro (M.T.L.); cgavrila@unitbv.ro (C.C.G.)

**Keywords:** chain drive, tensioning guides, friction, tests, polyamides

## Abstract

The development of polyamides has increased in recent years due to scientific research and the aim of the industry to find materials characterized by good frictional behavior at high temperatures, high loads and high speeds. The wide areas of application of polyamides in the mechanical contacts with relative motions offered new development opportunities for polyamides (PA), such as PA66, PA46 and PTFE mixed PA46. One of the applications of these polyamides in mechanical contacts with relative motions is the contact between the timing chains and the active part of the tensioning guides. For a comparison with a previous study of friction of PA66, PA46 and PTFE mixed PA46, performed on a pin-on-disk tribometer, this study is based on performing tests on the same materials, on the tensioning guide of a silent chain used as a timing chain in combustion engines, with lubricating conditions much closer to the application field. The diagrams comparatively present the variation of the percentage of frictional losses for PTFE mixed PA46, PA46 and PA66 polyamides mounted on tensioning guides in contact with chains. The results clearly show that the PTFE mixed PA46 polyamide has smaller frictional losses than the PA46 and the PA66 polyamides, but the influence of speed and tensioning is also important. A comparison with the previous study is also useful for making a decision on using a polyamide for the guide of a timing chain.

## 1. Introduction

One of the aims of developing new materials is to obtain better mechanical properties which ensure higher durability of the mechanical systems in which they are used. The durability, as one of the performance criteria which is considered in the design process of the mechanical systems, is strongly connected to the tribological properties (wear, friction) of the mechanical parts being in contact and in relative motion. One of the domains focused on development of new materials is represented by the polymers, with their subdomain, the polyamides. The development of this field is sustained by the necessity of finding new materials characterized by low friction, with application in areas such as mechanical transmissions, medical devices, the food industry, electronics and the chemical industry.

Polyamide blends with nanostructures of carbon are becoming popular. The chromium impurities’ influence on carbon nanotubes’ electron transport is presented in [1]. The authors of [2] studied the physical behavior of silicon carbide crystals and films from the phase transformation point of view, and the correlation with the physical materials’ behavior.

A study of the friction coefficient and wear properties of the polyamide blends (polyamide 66 (PA66) and polyphenylene sulfide (PPS)) is presented in [3]. Polyamide blends with the lowest wear depending on the adhesive ability of the PP are identified, and the frictional properties depend on the PA melting point.

The polyamide-type materials are implemented in the construction of the mechanical contacts with relative motion due to their high durability and low friction in the case of medium temperatures, high loads and high relative speeds. The influence of the addition of graphite to PA6 on its tribological properties, by using pin-on-disk tribological tests, is investigated in [4]. The conclusion shows that the tribological properties are enhanced by adding graphite.

The frictional properties of the polyamides are presented in the literature, based on tests measuring friction coefficients on tribometers for the polyamide on steel-type contacts, or measuring friction forces or friction torques in mechanical transmissions. All these measurements are achieved under different test conditions of load, speed and temperature. 

The tests performed on tribometers, depending on the devices which are used, follow two directions: one is represented by the ball-on-block-type contacts, where the relative motion is a linear reciprocating one, and the other is represented by the pin-on-disc-type contacts, where the relative motion is a continuous rotational one.

An experimental study of the frictional and wear properties of PA46 and PA46/aramid-fiber composites, under different velocities and loading conditions, was conducted in [5], on a pin-on-disc tribometer. It was identified that the PA46/aramid-fiber composite blends ensure the lowest friction coefficients and high steady wear rates at high loads and velocities, and the influence of the local temperature on the tested polyamides’ behavior was also studied. Another experimental study [6] using a tribometer presents results on the tribological behavior of the PA46 and the PA66 under rolling and sliding contact conditions.

The tests presented in [7,8] are performed under non-lubricated conditions, on tribometers equipped with a reciprocating module, for a contact between a steel-made ball and a PA66 polyamide plate. The friction coefficient is studied in [7], considering different loadings and sliding velocities. The results show that the friction coefficient decreases with the increase of the loading and increases with the increase of the speed, with values in the range 0.15–0.4. In [8], the value of the friction coefficient is increasing with the increase of the frequency, with values in the range 0.22–0.28.

For non-lubricated conditions, in [9], the test parameters are represented by the values of the normal forces between 50 and 250 N and the value of the velocity up to 0.1 m/s. The value of the friction coefficient for the PA66-made disk in contact with the steel-made pin is increasing with the increase of the force (between 0.15 and 0.23) and is decreasing with the increase of the speed. For the same range of the normal forces, in the case of lubricated conditions, the friction coefficient decreases with the increase of the force (between 0.05 and 0.06). The increase of the friction coefficient for the PA66 polyamide, with values between 0.2 and 0.35, with the increase of the product between the normal pressure and the speed, PV, is noticed in [10]. In this paper, the value of the friction coefficient was calculated by measuring the friction torque with a torque transducer.

In order to be used for automobile transmission, the development of new polyamides characterized by lower friction for temperatures higher than 100 °C is focused on the area of graphite, glass fiber or polytetrafluoroethylene (PTFE) mixed PA polyamides. Comparative results of the frictional properties for the PA66 and PA46 polyamides are presented in [11,12]. According to these papers, at high temperatures (210 °C), in the case of the contacts with steel-made materials, the PA46 polyamides are characterized by smaller friction coefficients than the PA66 polyamides. 

The literature also contains studies regarding the properties of the polyamides used in mechanical and automotive transmissions. According to [13], the PA6 and the graphite mixed PA6 have self-lubricating properties, which assure good frictional behavior combined with advantages such as a simple and economic manufacturing process. Good tribological properties of the PA-type polyamides are highlighted in [14,15].An application of the PA66 polyamides in the construction of the journal bearings is presented in [16]. Here, the tests show that the wear of the studied polyamides (PA66 and glass fiber MoS_2_-reinforced PA66) increases with the increase of the temperature, the pressure and the sliding velocity. 

Short carbon/PA66/PTFE hybrid composites are studied in [17], determining dry friction coefficients, showing a reduction of the friction coefficient and wear for certain concentrations in the mixture. 

In order to reduce the fuel consumption in automobiles, one of the research aims is to find solutions for timing chains characterized by low friction. According to this, theoretical models have been developed, such as that in [18], which allows predicting the friction loss of the timing chain under different conditions. It was found that a reduction of this sliding friction has a high influence on the global friction loss in the timing chain. The tensioning guide of the chain is made by a PA46 polyamide in order to obtain high efficiencies. 

The authors of [19] present a method used to precisely evaluate, by using tests, the friction losses in timing chains. According to the results, almost 25% of the energy losses are coming from the contact between the guide and the chain.

The authors of [20] studied the friction losses in the two guides of a chain drive transmission by considering different materials, depending on speed. According to the study, some optimizations of the materials used in the chain drive are proposed in order to reduce the friction. A solution used for reducing the friction in timing chains is presented in [21], by identifying a new polyamide PA46-type material which is applied on the active side of the tensioning guide. This material was a PTFE nanotube mixed PA46, similar to the one studied in the current paper. The authors present a study on the influence of the temperature, oil viscosity, oil age and surface roughness on the frictional losses from the guide/chain contact. The influence of load and speed is not presented. The influence of the PA66 and PA46 polyamide materials used for the construction of the tensioning guides in chain drive transmissions on the frictional losses is presented in [22]. The influence of load and speed on friction coefficients is studied on tribometers. It is highlighted that the PA46 polyamides have a better frictional behavior which ensures lower fuel consumptions and lower CO_2_ emissions.

The authors of [23] present an evaluation of the friction coefficients for the PTFE mixed PA46, the PA46 and the PA66 polyamides. The tests were performed for speeds between 0.025 and 2 m/s and medium pressures between 0.159 and 3.183 MPa. The conclusions of the paper highlight that the PTFE mixed PA46 has the smallest friction coefficients for all the testing conditions and a smaller sensitivity of the friction coefficient with the variation of the temperature, speed and local pressure. All the tests showed a decrease of friction coefficients with the increase of speed and pressure. In comparison with the current study, the friction coefficient measured on PTFE mixed PA46 in [23], at a speed of 2 m/s, 3.183 MPa medium pressure and 90 °C temperature, was around 0.06.

All of the discussions in [23] are based on tests performed on a pin-on-disk tribometer. Even with lubrication in an oil bath, these tests are far from the conditions of the case of the tensioning system of timing chains. On the tribometers, the speed is limited, due to limitations of the diameter of the disk. The lubricating conditions on the tribometer are proper for boundary lubrication but not for hydrodynamic lubrication. This is the reason why the present paper studies the frictional behavior of the PTFE mixed PA46, the PA46 and the PA66 polyamides in conditions much closer to the case of the contact between the active part of the tensioning guide (made from polyamides) and the timing chain drive used for combustion engines. Conclusions drawn from the comparison with the friction tests on the tribometer, for the same three types of polyamides, are presented for a better understanding of the influence of real lubricating conditions on the frictional behavior of the tested polyamides.

The literature offers results regarding tests which highlight the frictional properties of polyamides, but less on the frictional losses in chain drive transmissions (with or without guides) depending on speed and load. The aim of this paper is to evaluate the frictional losses, depending on speed and load, in a practical use of the polyamides as the active part of the tensioning guide used in a chain drive transmission. The frictional losses are highlighted comparatively for three types of polyamides: the PTFE mixed PA46, the PA46 and the PA66. The obtained results provide information on which type of polyamides should be used in tensioning guides for smaller frictional losses, and on the differences in friction losses in the case of different ranges of speed and normal load.

## 2. Materials and Methods

### 2.1. Testing Equipment

**Figure 1:**
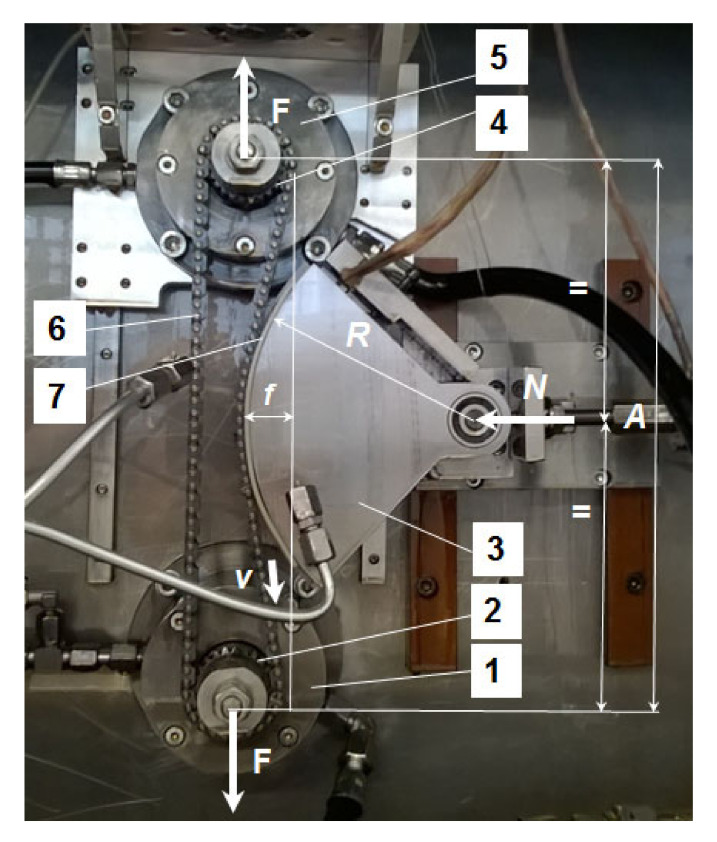
The main elements, dimensions and loads of the translational guiding system with a circular guide. (The components of the transmission are: 1—bearing box of the input shaft, 2—input chain sprocket, 3—circular guide, 4—output chain sprocket, 5—bearing box of the output shaft, 6—chain and 7—polyamide tape.)

The tests referring to the friction between the tensioning guide and the chain were performed on the test rig presented in [24], where more details on the rig and testing procedures are presented. The test rig is used for measuring friction losses in mechanical transmissions with a transmission ratio equal to 1 and can be equipped with a guiding system. The test rig is equipped with a torquemeter for torque measurement at the input shaft of the transmission, T. The measured torque represents the total amount of the resistant torques from the mechanical transmission which, according to the transmission’s typology, could be represented by the friction in bearings, the friction in the chain and the friction in the guide. Figure 1 presents the front view of the chain transmission mounted on the test rig, together with the tensioning guide, as it was used for the tests presented in this paper. 

The following parameters could be measured and controlled: the rotations at the driving sprocket, *n*, the tensioning force, *F*, of the chain drive transmission, which determines the push force, *N*, of the circular guide (see Figure 1), and the oil temperatures and pressures from the two flow circuits (in bearings and in the chain drive and the tensioning guide).

### 2.2. Testing Procedure

There are two mechanical configuration cases which allow performing measurements on the test rig: one without a tensioning guide and one with a tensioning guide.

In the case without a tensioning guide, the measured torque (*T*) consists of the resistant torque from the bearings and the resistant torque from the chain (with the transmission ratio equal to 1) and is measured depending on the rotational speed, *n*, at the driving sprocket and the tensioning force, *F* (see Figure 1), for constant temperature and pressures of the oil in the bearings and chain drive flow circuits. Mathematically, this can be expressed as:

*T = T_f_bearings+chain_ = T _fbearings_ + T _fchain_*. (1)

In the case where a tensioning guide is used, the measured torque contains the resistant torque from the bearings, the resistant torque from the chain (with the transmission ratio equal to 1) and the resistant torque from the guide friction. The torque is measured depending on the rotational speed, *n*, at the motor and the tensioning force, *F*, for a given position, *f*, of the tensioning guide (see Figure 1), for constant temperatures and pressures for the oil in the bearings and chain drive flow circuits. Mathematically, this can be expressed as:

*T = T_f_bearings+chain+guide_ = T _fbearings_ + T _fchain_ + T _fguide_*. (2)

Finally, the friction loss in the guide results as the difference between the torque measurements from the previous two tests:

*T_fguide_ = T_f_bearings+chain+guide_ − T_f_bearings + chain_*. (3)

The tests were repeated for one chain (C), with three samples (S1, S2 and S3) of each of the three guide materials (PA66, PA46 and PTFE PA46).

Table 1 presents the subject of testing (C—chain or C + guide, where the guide material is one of the three samples, S1, S2 and S3, of PA66, PA46 and PTFE PA46), the type of tests (R—running-in, M—measurements), controlled test parameters, number of repetitions and duration, in the order of testing.

At first, because the chain is new, a running-in type test (R) was performed on the chain without the tensioning guide. The test was achieved over a period of 50 h in constant test parameter conditions. The rotational speed of the driver sprocket is *n* = 1800 rpm, the tensioning force is *F* = 500 N, the oil in the bearings’ flow has a temperature *t* = 40–45 °C and the oil in the chain flow has a temperature *t* = 50–60 °C. The torque was measured during the running-in test, showing that the friction losses were stabilized. After the first 10 h of running-in, the measured torque dropped by approximately 10%, while during the following 40 h of running-in, the total drop of torque was 1%. This value assures that no important changes in chain friction took place in the following tests, which in total took another 50 h.

The next type of tests were measurement tests (M), performed on the chain without the tensioning guide. The tests were repeated 3 times. The measured torque, *T_f_bearings+chain_*, contains the friction from the bearings and from the chain and is highlighted depending on the rotational speed, *n*, and the tensioning force, *F*.

The third type of tests were performed with the circular tensioning guide mounted on the test rig. Three samples each of the PA66, PA46 and PTFE mixed PA46 polyamide tapes assembled on the tensioning guide were tested with the same chain. 

For each pair of chain/polyamide, the measurement tests (M) have been repeated 2 times and were preceded by 3 h running-in (R), dedicated to the new polyamides’ tapes, at a constant rotational speed *n* = 1800 rpm, a constant tensioning force *F* = 500 N, a temperature of the oil in the bearings flow of *t* = 55–65 °C and a temperature of the oil in the chain flow of *t* = 90–100 °C. For each pair of chain/polyamide, the tests, including running-in, took about 6 h.

All the measurements were achieved for constant functioning conditions (constant rotational speed, tensioning force, oil temperature) when the measured torque is stabilized.

### 2.3. Materials for Testing

The tests were achieved for a single-row, silent chain drive with a transmission ratio equal to 1, with 126 links and 8 mm pitch. The number of teeth of the sprockets is *z* = 23. The center distance is *A* = 300 mm, and the position of the tensioning guide in the horizontal direction is *f* = 25 mm; in the vertical direction, the guide is positioned at half of center distance *A* (see Figure 1). The temperature of the chain and guide lubrication oil is 100 °C ± 1%, similar to the running conditions in a combustion engine, while the temperature of the bearings’ lubrication oil is 60 °C ± 1%. The oil used for the lubrication is a 5W30 type, designed for combustion engines. 

Three types of polyamides (PA66, PA46 and PTFE mixed PA46) were tested, assembled as 2 mm thick tapes on the circular part of the tensioning guide, where the circular part has the radius *R* = 122 mm. The same three types of polyamides have been tested before on a pin-on-disk tribometer (disk made of polyamide), and the results [23] show friction coefficients depending on the type of polyamides, speed, pressure and oil temperature. In the tests presented in the current paper, the range of speed is larger and the conditions of lubrication, allowing possible hydrodynamic lubrication, depend on the application of polyamide on steel contact for the tensioning device of the timing chains.

The mechanical properties of the polyamides used as the active parts of the tensioning guides are presented as follows [23]: The PA66 polyamide was obtained by injection molding, unreinforced and heat-stabilized, with a humidity absorption of 2.5%, a tensile modulus of 3000 MPa, the deflection temperature at 1.8 MPa stress was 75 °C and the melting temperature was 260 °C [23]. The PA46 polyamide was obtained by injection molding, unreinforced and heat-stabilized, with a humidity absorption of 3.4%, a tensile modulus of 2800 MPa, the deflection temperature at 1.8 MPa stress was 190 °C and the melting temperature was 295 °C [23]. The PTFE mixed PA46 polyamide was heat-stabilized and friction-modified, with a humidity absorption of 3.2% and a tensile modulus of 3100 MPa. 

## 3. Results

### 3.1. Basics of Measurement Procedure

**Figure 2:**
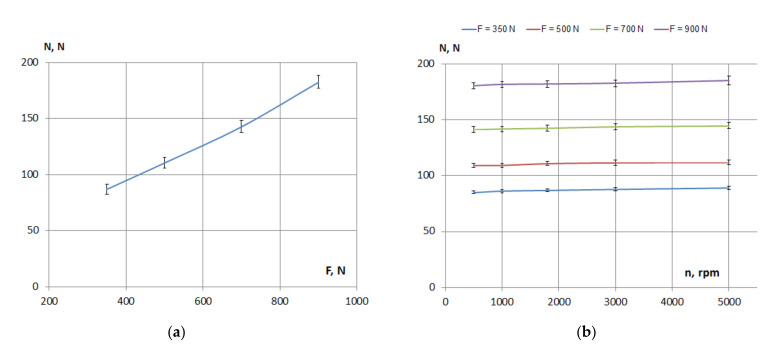
Pushing force of the tensioning guide depending on the tensioning force (**a**) and rotational speed (**b**).

The variation of the pushing force, *N* (see Figure 1), of the tensioning guide with the tensioning force, *F*, of the chain and with the rotational speed, *n*, is presented in Figure 2, for the measurements on one sample of PA66. According to Figure 2a, it can be assumed that it is a linear variation of the pushing force of the guide depending on the tensioning force of the chain. For an increase of the rotational speed from 500 to 5000 rpm (Figure 2b), the pushing force of the guide had a small increase, which as a percentage was up to 1.5%, determined by the centrifugal effect on the chain. The measured pushing force, *N*, is needed for evaluation of the medium pressure between the chain and the polyamide guide. For a normal pushing force in the range of 85–185 N, the calculated medium pressure is in the range 0.3–0.7 MPa, with possible higher values at the middle of the chain–guide contact and lower values at the sides.

**Figure 3:**
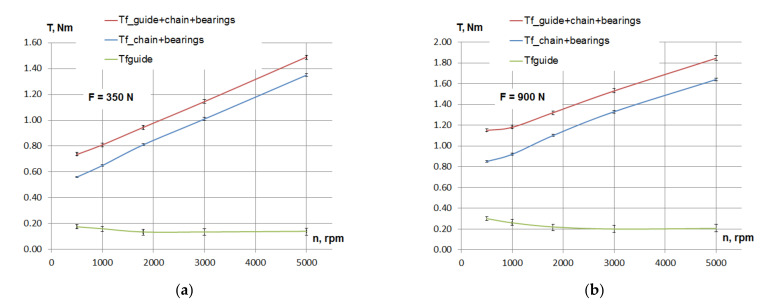
Basics of determining the friction losses in the contact between the guide and the chain: (**a**) tensioning of the chain *F* = 350 N and (**b**) tensioning of the chain *F* = 900 N.

Figure 3 highlights the basics of determining the friction losses in the contact between the guide and the chain for the PA66 guide material. The friction losses (as resistant friction torque) in the contact between the tensioning guide and the chain (*Tfguide*) are determined as extracting the friction torque in bearings and chain (*T_f_chain+bearings_*) from the friction torque in bearings, chain and guide (*T_f_guide+chain+bearings_*). The diagrams are plotted for two values of the tensioning force of the chain, depending on rotational speed.

For the repeated tests, the values are averaged. In the case of testing three samples of the same polyamides, the results are averaged once for each sample (from two measurements) and then averaged again for the three samples. The error bars presented in Figure 3 are considering all six measurements on PA66. The error bars on the calculated *T_fguide_* (see Equation (3)) are relatively large because they result from adding the errors of the *T_f_guide+chain+bearings_* and *T_f_chain+bearings_*.

The following diagrams show the friction losses as percentage values of friction torques relative to the minimum value of the friction torque from each diagram (*T*%*_min_* = 100%). Therefore,

*T*% = *T_x_/T_min_* 100 (4)

where *T_x_* is the current value of the torque and *T_min_* is the minimum value of the torque from each diagram. The errors are also calculated in percentages of T_min_.

The results are presented depending on the linear sliding speed, *v* (see Figure 1), of the chain on the guide, directly depending on the rotational speed, *n*. The reason is that these values are needed for the evaluation of friction losses depending on load and linear speed, for comparison with previous studies. The linear speed of the previous pin-on-disk tribometer tests [23] was in the range 0.025–2 m/s, so only the smallest value of our test, *v* = 1.5 m/s (*n* = 500 rpm), is in that range.

### 3.2. Linear Speed Influence on Friction Losses

**Figure 4:**
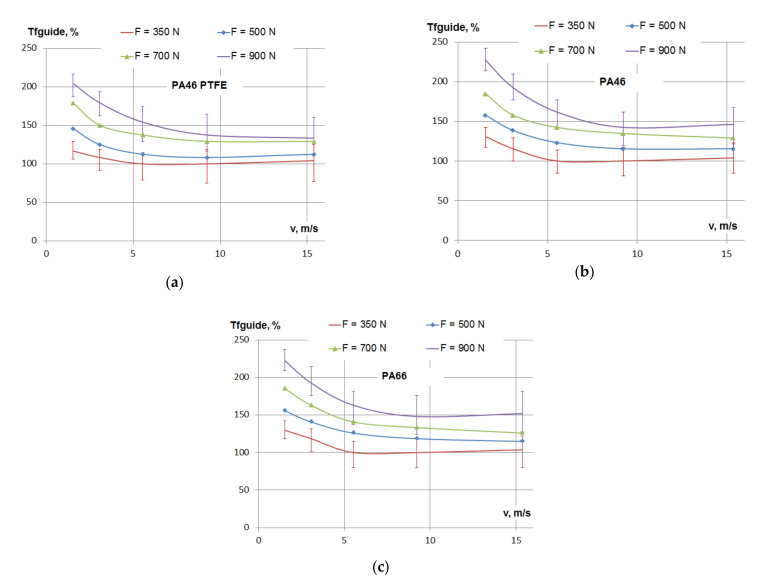
Friction losses in the contact between the guide and the chain depending on speed: (**a**) PTFE mixed PA46 polyamide, (**b**) PA46 polyamide and (**c**) PA66 polyamide.

Figure 4 presents the variation of the friction losses in the guide on chain contacts for PTFE mixed PA46, PA46 and PA66 polyamides, depending on linear speed, for different tensioning forces of the chain. The error bars are only presented on the top and bottom curves, in order to avoid crowding from overlapping. The other error bars are similar. This is also valid for the figures that follow.

In all cases, for linear speed in the range 1.5–5.5 m/s, the friction losses decreased with the increase of speed. For the smallest (*F* = 350 N) tensioning force, the decrease in friction was somewhere between 15%, for PTFE mixed PA46, and 30%, for PA66. In the same range of linear speed, for the highest (*F* = 900 N) tensioning force, the decrease in friction was somewhere between 20%, for PTFE mixed PA46, and 25%, for PA66 and PA46.

The decrease of friction losses continued up to the speed of 9 m/s, to more than a 50% drop for PTFE mixed PA46 and PA46, and 45% for PA66, in the case of the *F* = 900 N tensioning force. 

For high linear speed, in the range 10–15 m/s, the friction had no significant variation, especially for smaller loads. Due to errors of measurements, it is not clear if there is a continuous decrease, stabilization or increase.

### 3.3. PA Material Influence on Friction Losses

**Figure 5:**
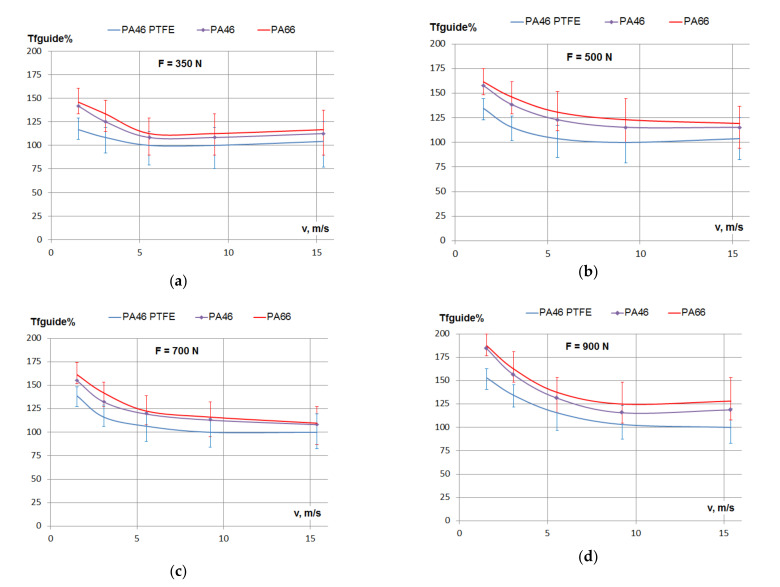
Friction losses in the contact between the guide and the chain depending on speed: (**a**) tensioning of the chain *F* = 350 N, (**b**) tensioning of the chain *F* = 500 N, (**c**) tensioning of the chain *F* = 700 N and (**d**) tensioning of the chain *F* = 900 N.

The same conclusions may be drawn according to Figure 5, which presents the variation of the percentage of frictional losses for the three types of polyamides, depending on the linear speed.

The resistant friction torque in the guide, calculated as a product between friction force in the guide and the radius of the chain sprocket, directly depends on the normal force on the guide (which directly depends on the tensioning force, see Figure 1) and on the average friction coefficient between the PA guide and the chain.

The comparison between friction losses in PTFE mixed PA46, PA46 and PA66 polyamides, from Figure 5, is actually a comparison between friction coefficients. It shows that PTFE mixed PA46 presented the smallest friction of the three. The maximum difference between PTFE mixed PA46 and PA46 was 21% at the lowest speed and smallest load. At higher speed and small to medium loads, the difference between PTFE mixed PA46 and PA46 was less than 10%. Friction losses in PA46 and PA66 were very close, but clearly PA46 had lower friction than PA66, with a maximum of 8%. This conclusion is in accordance with the pin-on-disk test results presented in [23], where the friction coefficient was measured for contacts between PTFE mixed PA46, PA46 and PA66 polyamides and a steel-made pin.

For the range of speed of 1.5–2 m/s and the range of medium pressure of 0.1–0.5 MPa, a comparison between the two studies showed that: The PTFE mixed PA46 friction coefficient was smaller than the PA46 friction coefficient by 15% according to [23], and by 20% according to the current study. The PA46 friction coefficient was smaller than the PA66 friction coefficient by less than 5% according to [23], and by 8% according to the current study. We do not have results for smaller linear speeds, where the timing chain only passes at the start, but according to [23], for a 0.025 m/s linear speed, the PTFE mixed PA46 friction coefficient is smaller than the PA46 friction coefficient by 30%. 

### 3.4. Tensioning Force Influence on Friction Losses

**Figure 6:**
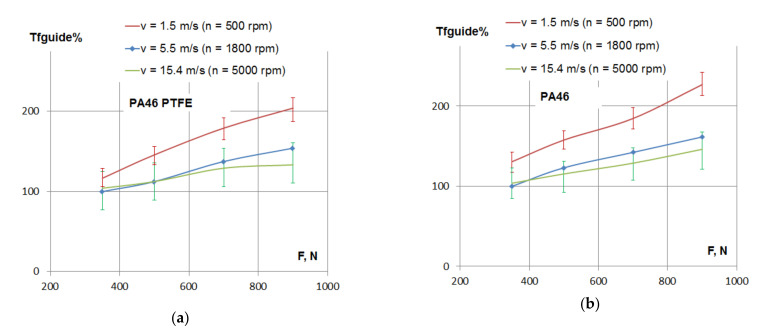

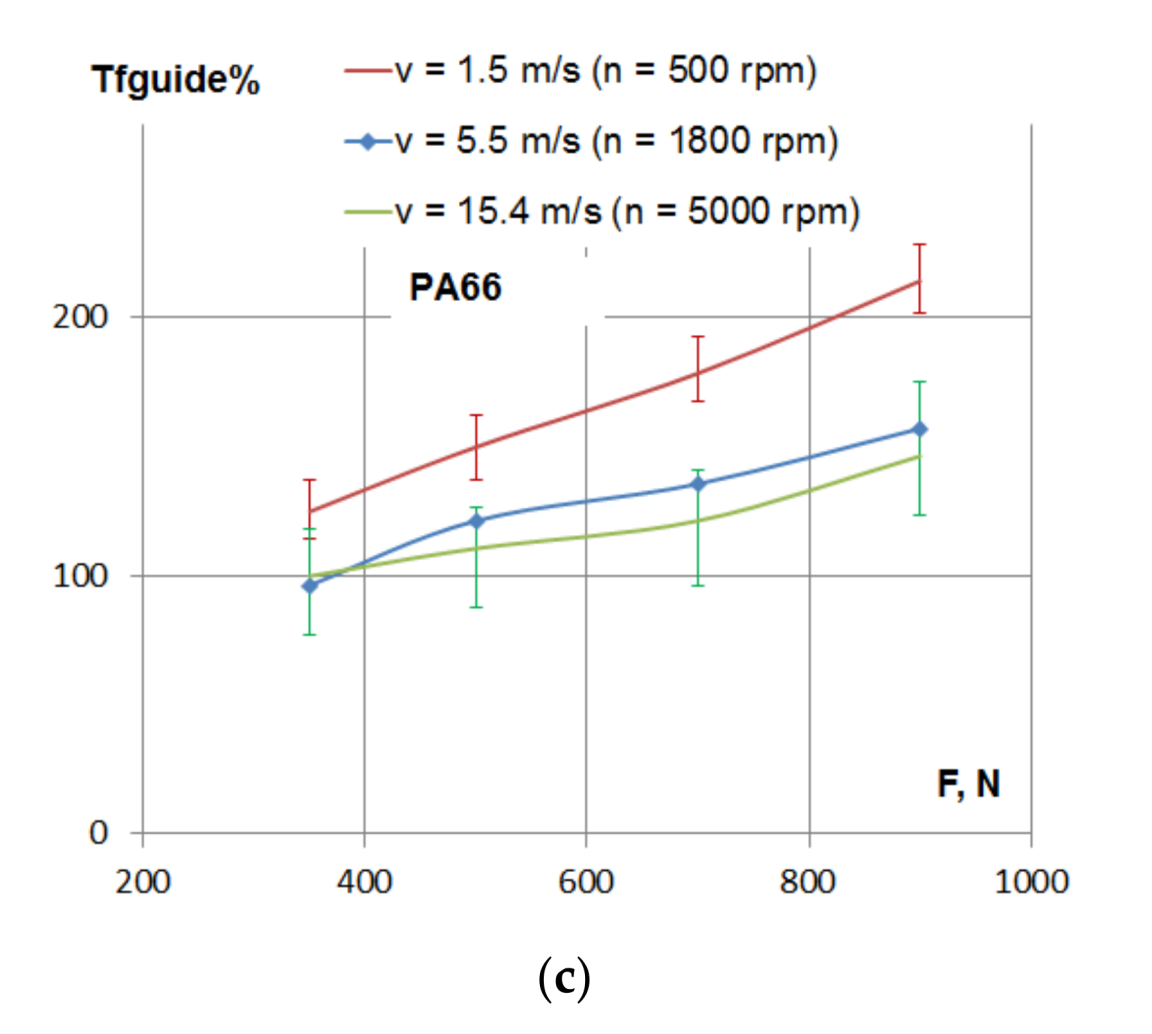
Friction losses in the contact between the guide and the chain depending on tensioning force: (**a**) PTFE mixed PA46 polyamide, (**b**) PA46 polyamide and (**c**) PA66 polyamide.

The variation of the percentage of frictional losses with the tensioning force of the chain is presented in Figure 6. For all the tested polyamides, the frictional losses increased with the increase of the tensioning force. 

This is absolutely normal, because the friction torque directly depends on the friction force and the friction force directly depends on the normal force. 

According to Figure 2a, with the increase of tensioning load, F, from 350 to 900 N (a 157% increase), the normal force, N, increased by 120%. According to Figure 6, with the increase of tensioning load in the same range, the maximum increase of friction losses were found for a lower speed (1.5 m/s): about 75% for all tested polyamides. For a higher speed (15.4 m/s), the same increase in load determined about a 50% increase of friction losses for PA46 and PA66, but only 35% for the PTFE mixed PA46 polyamide.

**Figure 7:**
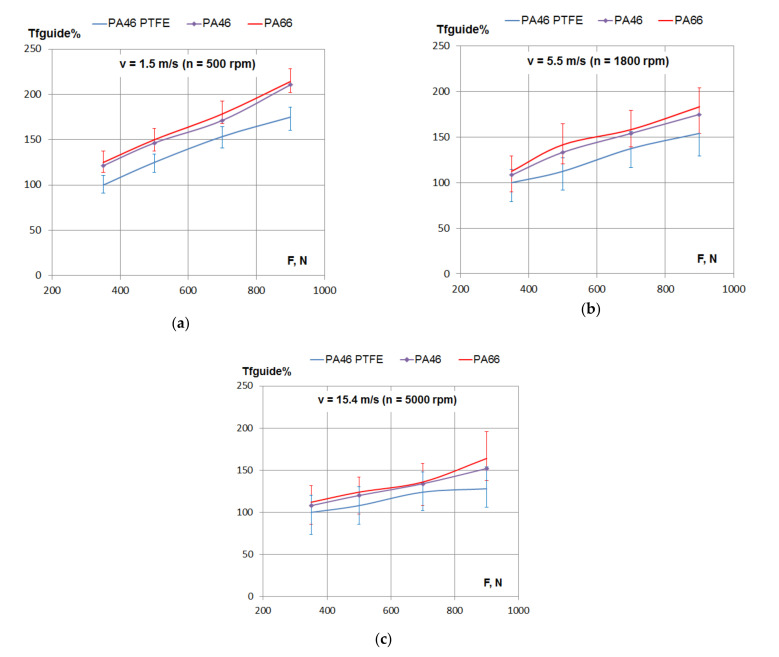
Friction losses in the contact between the guide and the chain depending on tensioning force: (**a**) linear speed *v* = 1.5 m/s, (**b**) linear speed *v* = 5.5 m/s and (**c**) linear speed *v* = 15.4 m/s.

The same conclusions may be drawn according to Figure 7, where the variation of the percentage of frictional losses is presented separately for three values of linear speed.

### 3.5. Power Losses and Friction Coefficient Depending on Tensioning Force and Speed

Power losses coming from the chain–guide friction result as a product between resistant friction torque in the guide and angular speed. Since the angular speed is constant (constant rotational speed) for each of the diagrams in Figure 7, it means that Figure 7 is an image of power losses, in percentage, depending on tensioning. For the normal running condition of the timing chain of combustion engines, which is medium to high speed (5.5 to 15.4 m/s) and small to medium tensioning (350 to 700 N), the difference in power losses between PA46 and PTFE mixed PA46 results in a range of 7% to 20%.

**Figure 8:**
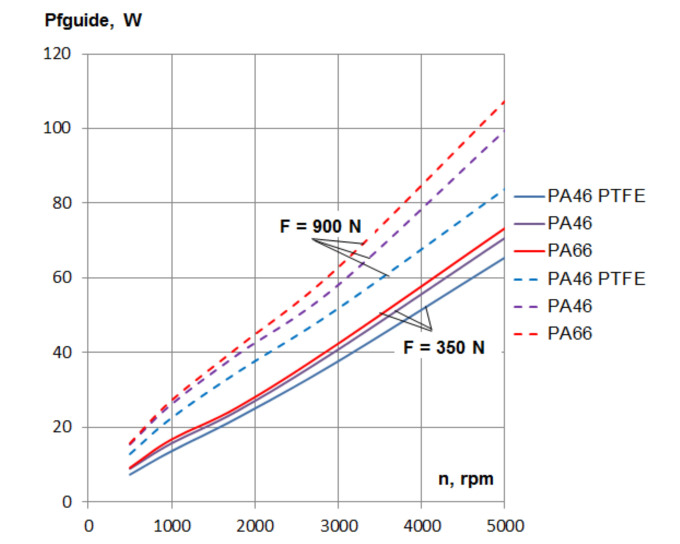
Power losses caused by friction in the guide, depending on rotational speed.

Figure 8 presents the calculated power losses caused by friction in the guide, *Pfguide*, depending on the rotational speed, for the three PA guide materials, for the lowest and highest tensioning of the tests. A comparison between using PA46 and PTFE mixed PA46, for a 5000 rpm rotational speed, showed a reduction of power losses of about 7 W in the case of 350 N tensioning and of about 15 W in the case of 900 N tensioning.

**Figure 9:**
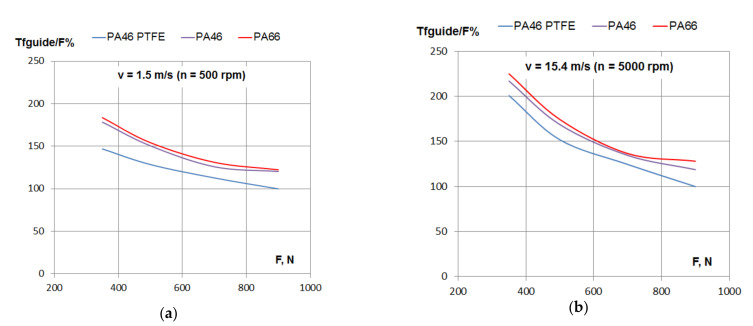
Ratio between friction losses and tensioning depending on tensioning force: (**a**) linear speed *v* = 1.5 m/s and (**b**) linear speed *v* = 15.4 m/s.

In order to evaluate the average friction coefficients between the guide and the chain depending on load, Figure 9 presents the ratio between the measured friction torque and tensioning force, in percentages, depending on the tensioning force. 

Figure 9 shows images of average chain–guide friction coefficients depending on load. The maximum value of the average chain–guide friction coefficient, calculated as friction force divided by normal force, resulted as 0.05 for PA66, for the lowest speed and smallest tensioning force. A minimum value of 0.015 resulted for PTFE mixed PA46, for a high linear speed and high tensioning force. These values are significantly smaller than the minimum friction coefficient from [23] based on the same polyamides, performed on a tribometer, which was 0.06. 

## 4. Discussion

The authors of [21] presented a study on friction torque in chains, measured on a full engine with three guides, with similar results to ours, about the ranking of the three types of polyamides, showing the same ranking results as [23].

The experimental studies on polyamides under lubricated conditions, performed on tribometers [10,13,15,22,23], show a decrease of the friction coefficient with linear speed. According to Stribeck’s curve, this indicates the presence of boundary friction, as mentioned in [22]. The diagrams from Figure 4 and Figure 5, presenting the influence of speed on friction, are similar to Stribeck’s curve, since, for constant tensioning, the friction torque and friction coefficient are directly dependent on each other. For a small linear speed, the decrease of friction with the increase of linear speed suggests that the chain–guide contact is subject to boundary friction. All the links of the chain in contact with the guide have the same linear speed, but not the same pressure. Pressure is higher at the middle of the guide and smaller on the links entering and exiting the guide. The results from [21] and [23] show a decrease of the friction coefficient with the increase of speed, with a trend of stabilization starting from a 1 m/s linear speed. The tests performed in this study (see Figure 5) showed that the decrease of friction continued up to a 5 m/s speed, where the stabilization began. Since boundary lubrication is the case in tribometer tests, in the case of guide on chain tests, with better lubrication, it is possible that mixed or hydrodynamic lubrication may occur, explaining the continuous drop of friction with higher speeds (mainly at the links of the chain entering the contact with the guide, where pressure is smaller, and where the oil is thrown on the guide). The smaller values of friction coefficients determined on the chain–guide testing, in comparison to measurements on tribometers, suggest the same explanation.

The experimental studies on polyamides under lubricated conditions, performed on tribometers [10,15,21,22,23], have shown a decrease of the friction coefficient with increasing pressure, which was also shown by the results presented in Figure 9. This is opposite to Stribeck’s curve for boundary friction and is explained [22,23] by adhesion-dominated friction on polyamides. 

## 5. Conclusions

The PTFE mixed PA46 polyamide had smaller frictional losses than PA46, which in turn had smaller frictional losses than the PA66 polyamides. This is the same ranking obtained from tests carried out on tribometers, but the chain–guide tests performed here showed smaller differences and less friction. 

In order to choose one of the three PA materials for the guide of the chain drive, the value of the gain in power by the reduction of losses presented in Figure 8 should be useful.

Another important aspect that should further be investigated is the wear comparison between the different types of polyamides, since the other studies showed that PA66 has smaller wear than PA46, and PTFE mixed PA46 has the highest wear in lubricated conditions.

## Figures and Tables

**Table 1:** Testing order.

No.	Subject	Type	*F*, *N*	*n*, rpm	t, °C	Repetitions	Duration, h
1	C	R	500	1800	50–60	1	50
2	C	M	350	500/800/1500/3000/5000	100	3	1.5
3			500	500/800/1500/3000/5000	100	3	1.5
4			700	500/800/1500/3000/5000	100	3	1.5
5			900	500/800/1500/3000/5000	100	3	1.5
6	C + S1 PA66	R	500	1500	100	1	3
7	C + S1 PA66	M	350	500/800/1500/3000/5000	100	2	1
8			500	500/800/1500/3000/5000	100	2	1
9			700	500/800/1500/3000/5000	100	2	1
10			900	500/800/1500/3000/5000	100	2	1
11	C + S2 PA66	R	500	1500	100	1	3
12	C + S2 PA66	M	350	500/800/1500/3000/5000	100	2	1
13			500	500/800/1500/3000/5000	100	2	1
14			700	500/800/1500/3000/5000	100	2	1
15			900	500/800/1500/3000/5000	100	2	1
16	C + S3 PA66	R	500	1500	100	1	3
17	C + S3 PA66	M	350	500/800/1500/3000/5000	100	2	1
18			500	500/800/1500/3000/5000	100	2	1
19			700	500/800/1500/3000/5000	100	2	1
20			900	500/800/1500/3000/5000	100	2	1
21	C + S1 PA46	R	500	1500	100	1	3
22	C + S1 PA46	M	350	500/800/1500/3000/5000	100	2	1
23			500	500/800/1500/3000/5000	100	2	1
24			700	500/800/1500/3000/5000	100	2	1
25			900	500/800/1500/3000/5000	100	2	1
26	C + S2 PA46	R	500	1500	100	1	3
27	C + S2 PA46	M	350	500/800/1500/3000/5000	100	2	1
28			500	500/800/1500/3000/5000	100	2	1
29			700	500/800/1500/3000/5000	100	2	1
30			900	500/800/1500/3000/5000	100	2	1
31	C + S3 PA46	R	500	1500	100	1	3
32	C + S3 PA46	M	350	500/800/1500/3000/5000	100	2	1
33			500	500/800/1500/3000/5000	100	2	1
34			700	500/800/1500/3000/5000	100	2	1
35			900	500/800/1500/3000/5000	100	2	1
36	C + S1 PA46PTFE	R	500	1500	100	1	3
37	C + S1 PA46PTFE	M	350	500/800/1500/3000/5000	100	2	1
38			500	500/800/1500/3000/5000	100	2	1
39			700	500/800/1500/3000/5000	100	2	1
40			900	500/800/1500/3000/5000	100	2	1
41	C + S2 PA46PTFE	R	500	1500	100	1	3
42	C + S2 PA46PTFE	M	350	500/800/1500/3000/5000	100	2	1
43			500	500/800/1500/3000/5000	100	2	1
44			700	500/800/1500/3000/5000	100	2	1
45			900	500/800/1500/3000/5000	100	2	1
46	C + S3 PA46PTFE	R	500	1500	100	1	3
47	C + S3 PA46PTFE	M	350	500/800/1500/3000/5000	100	2	1
48			500	500/800/1500/3000/5000	100	2	1
49			700	500/800/1500/3000/5000	100	2	1
50			900	500/800/1500/3000/5000	100	2	1

## Data Availability

Restrictions apply to the availability of these data. Absolute data values are subject to a non-disclosure agreement with a third party. The relative data values, presented in the paper are available from the authors with the permission of the third party.

## References

[1] Repetsky P., Vyshyvana I.G., Nakazawa Y., Kruchinin S.P., Bellucci S. (2019). Electron transport in carbon nanotubes with adsorbed chromium impurities. Materials.

[2] Vlaskina S., Kruchinin S., Rodionov V. (2016). Nanostructures in silicon carbide crystals and films. Int. J. Mod. Phys. B.

[3] Zhaobin C., Tongsheng L., Yuliang Y., Xujun L., Renguo L. (2004). Mechanical and tribological properties of PA/PPS blends. Wear.

[4] Kumar S.S., Kanagaraj G. (2016). Investigation on mechanical and tribological behaviors of PA6 and graphite-reinforced PA6 polymer composites Arab J. Sci. Eng..

[5] Gordon D.H., Kukureka S.N. (2009). The wear and friction of polyamide 46 and polyamide 46/aramid-fibre composites in sliding–rolling contact. Wear.

[6] Wilson B.S. (2012). Traction and Wear Evaluation of a Number of Plastic Materials and Greases under Combined Rolling and Sliding Contact Conditions. Master’s Thesis.

[7] Shin M.W., Kim S.S., Jang H. (2011). Friction and wear of polyamide 66 with different weight average molar mass. Tribol. Lett..

[8] Haixia H., Sirong Y., Mingyu W., Kaixin L. (2009). Tribological behavior of polyamide 66-based binary and ternary composites. Polym. Eng. Sci..

[9] Kozma M. (2005). Effect of incorporated lubrication on the tribological properties of polyamides. Ann. Univ. Dunarea De Jos Galati.

[10] Shibata K., Yamaguchi T., Kishi M., Hokkirigawa K. (2015). Friction and wear behavior of polyamide 66 composite filled with rice bran ceramics under a wide range of PV values. Tribol. Online.

[11] Chiu F.G., Kao G.F. (2012). Polyamide 46/multi-walled carbon nanotube nanocomposites with enhanced thermal, electrical and mechanical properties. Composites.

[12] Chiu F.G., Fu S.W., Chuang W.T., Sheu H.S. (2008). Fabrication and characterization of polyamide 6.6/organomontmorillonite nanocomposites with and without a maleated polyolefin elastomer as a toughener. Polymer.

[13] Unal H., Mimaroglu A. (2012). Friction and wear performance of polyamide 6 and graphite and wax polyamide 6 composites under dry sliding conditions. Wear.

[14] Fasahat F., Dastjerdi R., Mojtahedi M.R.M., Hoseini P. (2015). Wear properties of high speed spun multi-component PA6 nanocomposite fabrics; abrasion resistance mechanism of nanocomposites. Wear.

[15] Zhang X.R., Pei X.Q., Wang Q.H., Wang T.M., Chen S.B. (2015). The friction and wear properties of carbon nanotubes/graphite/carbon fabric reinforced phenolic polymer composites. Adv. Compos. Mater..

[16] Mehnmet T.D., Hayrettin D., Rifat Y. (2013). Investigation of temperature effects on tribological properties of glass fiber and MoS2 reinforced PA6.6 and PA66 journal bearings. J. Mater. Sci. Eng..

[17] Wei R. (2014). Tribological properties of short carbon/PA/PTFE hybrid composites under dry sliding conditions. J. Thermoplast. Compos. Mater..

[18] Sakaguchi M., Yamada S., Seki M., Koiwa Y., Yamauchi T., Wakabayashi T. (2012). Study on reduction of timing chain friction using multi-body dynamics. Proceedings of the SAE 2012 World Congress & Exhibition.

[19] Hyakutake T., Inagaki M., Matsuda M., Hakamada N., Teramachi Y. (2001). Measurement of friction in timing chains. J. Soc. Automot. Eng. Jpn..

[20] Fink T., Bodestein H. (2011). Potential for reducing friction in chain drives. MTZ.

[21] Meuwissen M., Van Ruiten J., Besseling T., Van Sluijs R., Broda M., Pearce B., O’Shea F.I. (2017). Lowering friction in timing chain drive systems by tuning tensioner materials. SAE Int. J. Fuels Lubr..

[22] Van Ruiten J., Proost R., Meuwissen M. (2012). How the Choise of the Polyamide Type in Timing Chains Tensioning Systems Affects the CO2 Emissions and Fuel Economy of Internal Combustion Engines.

[23] Lates M.T., Velicu R.G., Gavrila C.C. (2019). Temperature, pressure and velocity influence on the tribological properties of PA66 and PA46 polyamides. Materials.

[24] Lates M.T., Velicu R.G., Jurj L. (2020). Influence of pitch and exploitation on the frictional behavior of the silent chains. Proc. Inst. Mech. Eng. Part D J. Automob. Eng..

